# How gut microbes affect Parkinson’s disease: an editorial

**DOI:** 10.1097/MS9.0000000000003098

**Published:** 2025-03-27

**Authors:** Rabia Nasir, Abdul Haseeb, Muhammad Saqlain Mustafa, Abdullah Mussarat, Laiba Ali, Muhammad Ashir Shafique, Khabab Abbasher Hussien Mohamed Ahmed

**Affiliations:** a Department of Surgery, Jinnah Sindh Medical University, Karachi, Pakistan; b Faculty of Medicine, University of Khartoum, Khartoum, Sudan

## Introduction

Parkinson’s disease (PD) is a progressive neurodegenerative disorder characterized by the accumulation of misfolded alpha-synuclein proteins in dopaminergic neurons of the basal ganglia, which primarily govern body movements. Key symptoms include resting tremors, postural instability, bradykinesia, and rigidity. In addition to motor function, individuals with PD often experience impaired gastric function, leading to issues such as nausea, vomiting, drooling, difficulty swallowing, bloating, and constipation. Studies conducted in animal models have indicated an imbalance in gut microbes, marked by an overabundance of opportunistic pathogens. This imbalance triggers systemic inflammation, particularly neuroinflammation, leading to neuronal degeneration, and ultimately, the onset of PD. These findings have given rise to the concept of the microbiome-gut-brain axis, which proposes a two-way relationship between gut microbes and the brain^[1]^. Braak’s theory further supports this idea, proposing that these microorganisms enter the gastrointestinal tract and subsequently advance into the brain via the vagus nerve, contributing to the development of PD^[2–4]^. Among the microorganisms found to be elevated in this disease are *Escherichia coli, Streptococcus, Shigella, Proteus*, and *Enterococcus*^[5–7]^. This study aims to investigate the underlying mechanisms of the disease and pave the way for innovative treatment possibilities in the future.

**HIGHLIGHTS**

- Gut microbes’ imbalance in Parkinson’s disease leads to systemic inflammation, including neuroinflammation and neuronal degeneration.
- The microbiome-gut-brain axis proposes a bidirectional relationship between gut microbes and the brain in Parkinson’s disease.
- Evolving therapeutic strategies include dietary modifications and the potential use of probiotics, prebiotics, symbiotics, and postbiotics to harness the therapeutic potential of gut microbes.
- Further research is required to establish the efficacy of these advancements in managing Parkinson’s disease.
- Understanding the connection between gut microbes and Parkinson’s disease offers new treatment avenues for this challenging condition.

## Pathogenesis related to gut-brain axis

Understanding the intricate connection between gut microbes and PD offers valuable insights into the pathogenesis of this condition, potentially opening new avenues for therapeutic interventions and disease management. Numerous investigations involving both humans and animals have yielded valuable insights into the microbiota-gut-brain axis^[8]^. Studies conducted on humans using imaging techniques have indicated that in certain instances of PD, alpha-synuclein protein may propagate from the gut to the brain^[9,10]^. Similarly, experiments in animal models have demonstrated that surgically removing the vagus nerve, which is responsible for connecting the gut to the brain, reduces the risk of PD^[11]^. In the quest to better comprehend the bacterial diversity within the gut, scientists are employing a novel approach known as “shotgun metagenomic sequencing.” This technique allows the identification of genera and species of gut microbes. It involves breaking down the DNA of all microorganisms into smaller fragments, which are then sequenced and analyzed. This process helps to gain a deeper understanding of the species and function of the genes encoded by these microbes^[12]^. Researchers have utilized shotgun metagenomic techniques to study focal samples from patients with PD and neurologically healthy individuals. The findings revealed that 30% of microbial species and genera were notably different between PD patients and healthy individuals. Additionally, fecal markers such as zonulin and alpha-1 antitrypsin, indicating increased intestinal barrier permeability, were elevated in patients with PD^[13]^.

Moreover, upon examining the brains, cerebrospinal fluid, and blood of patients with PD, scientists discovered heightened levels of inflammatory markers, including IL1, IL2, IL6, IL10, TNF, and CRP. These findings clearly indicate signs of inflammation^[14]^. Furthermore, studies have identified an intestinal inflammation marker called calprotectin, which is also elevated in PD patients^[15]^. Collectively, this evidence, along with ongoing research, strongly suggests that PD originates in the gut before advancing toward the brain, ultimately leading to neurodegeneration.

## Intestinal microbial changes in human PD

Results from various studies investigating the gut microbiota in PD reveal specific microbial changes, such as decreased Lachnospiraceae^[16]^, reduced Prevotellaceae^[17]^, elevated *Anaerotruncus* sp., *Clostridium* XIVa, Lachnospiracae^[18]^, higher Pasteurellaceae^[19]^, decreased Bifidobacterium, increased *Butyricicoccus* and *Clostridium* XlVb, and *Escherichia*/*Shigella*^[20]^, as well as elevated *Lactobacillus, Christensenellaceae*, and decreased Lachnospiraceae^[21]^, and increased Enterobacteriaceae^[22]^. These microbial alterations are associated with clinical outcomes, including disease duration, motor symptom severity, cognitive impairment, and non-motor symptoms, providing potential biomarkers and therapeutic targets in PD research.

## Current treatment options

PD affects a substantial global population; however, a definitive cure remains elusive. The primary therapeutic approach involves the use of anti-Parkinson medications such as Levodopa, Carbidopa, Madopar, and Sirelin. However, these medications have inherent limitations and their efficacy tends to diminish over time. Evidence has suggested that gut microbiota can influence levodopa kinetics, and conversely, drugs administered for PD can influence gut microbiota composition. Prebiotics can increase levels of short-chain fatty acid-producing bacteria and decrease pro-inflammatory species, with positive effects on clinical symptoms and levodopa kinetics. Different formulations of probiotics showed beneficial outcomes on constipation, with some of them improving dopamine levels; however, the most effective dosage duration and long-term effects of these treatments remain unknown^[23]^. Nonpharmacological interventions have also shown promise in the management of Parkinson’s. Research has demonstrated the usefulness of certain exercises, as well as therapies, such as speech and occupational therapies, in addressing both motor and non-motor symptoms of the condition. In addition to established treatments, ongoing clinical trials are investigating potential alternative options. These trials explored the effects of substances such as nicotine and caffeine, as well as the drug isradipine. Furthermore, active and passive immunizations against alpha-synuclein, along with inhibitors of its aggregation, are under investigation^[24]^. Although a definitive cure has not been found, the combination of current medications, non-pharmacological approaches, and ongoing research offers hope for improving the management and treatment of Parkinson’s, ultimately enhancing the quality of life for those affected by the condition.

## Evolving treatment paradigm for Parkinson’s disease

Recent advancements in therapeutic approaches for PD have capitalized on the understanding of gut microbes and their potential impact on the condition. One notable avenue involves the use of dietary modifications. Researchers have shown the significance of a ketogenic diet as a potential treatment that can reduce inflammation and modulate intestinal microbes^[25]^. Moreover, evidence suggests that adopting healthy dietary habits, such as consuming fruits, vegetables, and omega-3 fatty acids, and practicing calorie restriction, has neuroprotective properties. Such dietary choices have been associated with a lower risk and reduced severity of Parkinson’s. Omega-3 fatty acids, in particular, possess anti-inflammatory properties, mitigating the risk of oxidative stress and consequently reducing alpha-synuclein accumulation^[26]^. In addition to dietary interventions, various approaches involving gut microbes have been investigated, with some being in the clinical trial phase. Probiotics, psychotics, symbiotics, prebiotics, and postbiotics have shown therapeutic potential against Parkinson’s. Among these, lactic acid bacteria, such as Lactobacillus, Streptococcus, and Bifidobacterium, are commonly employed as probiotics. Studies have demonstrated that a combination of *L. acidophilus* and *B. subtilis* can alleviate bloating and abdominal pain in patients with PD^[27]^. Prebiotics, primarily dietary fiber, play a crucial role in preserving the health of the central nervous system. Symbiotics aid in restoring a healthy balance of gut microbes and improving gastrointestinal function in individuals with PD. Postbiotics have been shown to be effective owing to their anti-inflammatory, immunomodulatory, and antioxidative properties^[28]^.

These novel therapeutic advances offer promising possibilities for the management and treatment of PD by harnessing the potential of gut microbes and dietary interventions. However, further research and clinical trials are required to fully understand and validate their efficacy.

## Conclusions

PD impacts millions of individuals worldwide. Evidence strongly indicates that gut dysbiosis and opportunistic pathogens play critical roles in the onset and progression of PD. Furthermore, evidence suggests that adopting healthy dietary habits, such as consuming fruits, vegetables, and omega-3 fatty acids, has neuroprotective properties. As a result, researchers are now directing their attention toward gut health and the intricate relationship between gut microbes and PD. This study aims to deepen our understanding of the underlying mechanisms of the disease and pave the way for innovative treatment possibilities in the future.

## Data Availability

Data availability does not apply to this article as no new data were created or analyzed in this study.
